# Windschitl type approximation formulas for the gamma function

**DOI:** 10.1186/s13660-018-1870-0

**Published:** 2018-10-05

**Authors:** Zhen-Hang Yang, Jing-Feng Tian

**Affiliations:** 1 College of Science and Technology, North China Electric Power University, Baoding, P.R. China; 2 Department of Science and Technology, State Grid Zhejiang Electric Power Company Research Institute, Hangzhou, P.R. China

**Keywords:** 41A10, 26A48, 33B10, 26A51, Gamma function, Windschitl type approximation formula, Monotonicity, Convexity, Inequality

## Abstract

In this paper, we present four new Windschitl type approximation formulas for the gamma function. By some unique ideas and techniques, we prove that four functions combined with the gamma function and Windschitl type approximation formulas have good properties, such as monotonicity and convexity. These not only yield some new inequalities for the gamma and factorial functions, but also provide a new proof of known inequalities and strengthen known results.

## Introduction

For $x>0$, the classical Euler’s gamma function Γ and psi (digamma) function *ψ* are defined by

1.1 $$ \Gamma ( x ) = \int_{0}^{\infty }t^{x-1}e^{-t}\,dt \quad \text{and} \quad \psi ( x ) =\frac{\Gamma^{\prime } ( x ) }{ \Gamma ( x ) }, $$

respectively. The derivatives $\psi^{\prime }$, $\psi^{\prime \prime }$, $\psi^{\prime \prime \prime },\dots $ are known as polygamma functions. The gamma function has various important applications in many branches of science. For this reason, scholars strive to find various better approximations for the factorial or gamma function by using different ideas and techniques, for instance, Ramanujan [1, p. 339], Burnside [2], Gosper [3], Alzer [4], Shi et al. [5], Batir [6, 7], Mortici [8–12], Nemes [13, Corollary 4.1], [14], Qi et al. [15, 16], Feng and Wang [17], Chen [18–21], Yang et al. [22–25], Lu et al. [26–28], Xu et al. [29]. Some properties of the remainders of certain approximations for the gamma function can be found in [4, 16, 23, 30–35].

In this paper, we are interested in Windschitl’s approximation formula (see [36]) given by

1.2 $$ \Gamma ( x+1 ) \thicksim W_{0} ( x ) =\sqrt{2 \pi x} \biggl( \frac{x}{e} \biggr) ^{x} \biggl( x\sinh \frac{1}{x} \biggr) ^{x/2},\quad\text{as }x\rightarrow \infty . $$

As shown in [21, Eq. (3.18)], the rate of Windschitl’s approximation $W_{0} ( x ) $ converging to $\Gamma ( x+1 ) $ is like $x^{-5}$ as $x\rightarrow \infty $, and like $x^{-7}$ if one replaces $W_{0} ( x ) $ with

1.3 $$ W_{1} ( x ) =\sqrt{2\pi x} \biggl( \frac{x}{e} \biggr) ^{x} \biggl( x\sinh \frac{1}{x}+\frac{1}{810x^{6}} \biggr) ^{x/2} $$

by an easy check. These show that $W_{0} ( x ) $ and $W_{1} ( x ) $ are more accurate approximations for the gamma function. In 2009, Alzer [37] proved that for all $x>0$,

1.4 $$\begin{aligned} \sqrt{2\pi x} \biggl( \frac{x}{e} \biggr) ^{x} \biggl( x\sinh \frac{1}{x} \biggr) ^{x/2} \biggl( 1+\frac{\alpha }{x^{5}} \biggr) &< \Gamma ( x+1 ) \\ &< \sqrt{2\pi x} \biggl( \frac{x}{e} \biggr) ^{x} \biggl( x\sinh \frac{1}{x} \biggr) ^{x/2} \biggl( 1+\frac{\beta }{x^{5}} \biggr) \end{aligned}$$

with the best possible constants $\alpha =0$ and $\beta =1/1620$. Recently, Lu, Song and Ma [27] extended Windschitl’s formula to an asymptotic expansion:

1.5 $$ \Gamma ( n+1 ) \thicksim \sqrt{2\pi n} \biggl( \frac{n}{e} \biggr) ^{n} \biggl[ n\sinh \biggl( \frac{1}{n}+\frac{a_{7}}{n ^{7}}+ \frac{a_{9}}{n^{9}}+\frac{a_{11}}{n^{11}}+\cdots \biggr) \biggr] ^{n/2} $$

as $n\rightarrow \infty $ with $a_{7}=1/810,a_{9}=-67/42525,a_{11}=19/8505, \dots $, and proved that there exists an *m* such that, for every $x>m$, the double inequality

1.6 $$ \biggl[ x\sinh \biggl( \frac{1}{x}+\frac{1}{810x^{7}}-\frac{67}{42525x ^{9}} \biggr) \biggr] ^{x/2}< \frac{\Gamma ( x+1 ) }{\sqrt{2 \pi x} ( x/e ) ^{x}}< \biggl[ x\sinh \biggl( \frac{1}{x}+\frac{1}{810x ^{7}} \biggr) \biggr] ^{x/2} $$

holds. An explicit formula for determining the coefficients of $n^{-k}$ ($n\in \mathbb{N}$) was given in [19, Theorem 1] by Chen. Another asymptotic expansion

1.7 $$ \Gamma ( x+1 ) \thicksim \sqrt{2\pi x} \biggl( \frac{x}{e} \biggr) ^{x} \biggl( x\sinh \frac{1}{x} \biggr) ^{x/2+\sum _{j=0}^{\infty }r_{j}x^{-j}},\quad \text{as }x\rightarrow \infty , $$

was presented in the same paper [19, Theorem 2].

Let us consider the four new Windschitl type approximation formulas, as $x\rightarrow \infty $, which are

1.8 $$\begin{aligned}& \Gamma ( x+1 ) \thicksim \sqrt{2\pi x} \biggl( \frac{x}{e} \biggr) ^{x} \biggl( x\sinh \frac{1}{x} \biggr) ^{x/2}\exp \biggl( \frac{1}{1620x^{5}} \biggr) :=W_{01} ( x ) , \end{aligned}$$

1.9 $$\begin{aligned}& \Gamma ( x+1 ) \thicksim \sqrt{2\pi x} \biggl( \frac{x}{e} \biggr) ^{x} \biggl( x\sinh \frac{1}{x} \biggr) ^{x/2}\exp \biggl( \frac{1}{1620x^{5}}-\frac{11}{18{,}900x^{7}} \biggr) :=W_{02} ( x ) , \end{aligned}$$

1.10 $$\begin{aligned}& \Gamma ( x+1 ) \thicksim \sqrt{2\pi x} \biggl( \frac{x}{e} \biggr) ^{x} \biggl( x\sinh \frac{1}{x} \biggr) ^{x/2} \biggl( 1+ \frac{1}{1620x^{5}} \biggr) :=W_{01}^{\ast } ( x ) , \end{aligned}$$

1.11 $$\begin{aligned}& \Gamma ( x+1 ) \thicksim \sqrt{2\pi x} \biggl( \frac{x}{e} \biggr) ^{x} \biggl( x\sinh \frac{1}{x} \biggr) ^{x/2} \biggl( 1+ \frac{1}{1620x^{5}}-\frac{11}{18{,}900x^{7}} \biggr) =W_{02}^{\ast } ( x ) . \end{aligned}$$

The aim of this paper is, by investigating the monotonicity and convexity of the functions

$$ x\mapsto \ln \Gamma ( x+1 ) -\ln F ( x ),\quad\text{where }F=W_{01},W_{02},W_{01}^{\ast },W_{02}^{\ast }, $$

to establish some new sharp inequalities between the gamma function $\Gamma ( x+1 ) $ and Windschitl’s approximation formula $W_{0} ( x ) $. As a by-product, a concise proof of Alzer inequalities (1.4) is presented, and a strengthening for Lu et al.’s inequalities (1.6) is given.

The rest of this paper is organized as follows. In Sect. 2, three lemmas are given, which are crucial to the proofs of our results. In Sect. 3, five monotonicity and convexity results for the functions constructed from the gamma function and Windschilt’s formula are proved. Some new inequalities between the gamma or factorial functions with Windschilt’s formula are established in Sect. 4. In Sect. 5, numeric comparisons of several better approximation formulas are presented.

## Lemmas

To prove our results, we need three lemmas as follows.

### Lemma 1

*The inequalities*

2.1 $$\begin{aligned}& x\frac{x^{2}+\frac{71}{84}}{x^{4}+\frac{13}{14}x^{2}+\frac{27}{560}}< \psi^{\prime } \biggl( x+\frac{1}{2} \biggr) , \end{aligned}$$

2.2 $$\begin{aligned}& x\frac{x^{4}+\frac{227}{66}x^{2}+\frac{4237}{2640}}{x^{6}+ \frac{155}{44}x^{4}+\frac{329}{176}x^{2}+\frac{375}{4928}}< \psi^{ \prime } \biggl( x+\frac{1}{2} \biggr) < \frac{1}{x}\frac{x^{4}+ \frac{67}{36}x^{2}+\frac{256}{945}}{x^{4}+\frac{35}{18}x^{2}+ \frac{407}{1008}} \end{aligned}$$

*hold for* $x>0$.

### Proof

The inequality (2.1) was proved in [38, Remark 2.2].

Let

$$\begin{aligned}& g_{1} ( x ) =\psi^{\prime } \biggl( x+\frac{1}{2} \biggr) - \frac{1}{x}\frac{x^{4}+\frac{67}{36}x^{2}+\frac{256}{945}}{x^{4}+ \frac{35}{18}x^{2}+\frac{407}{1008}}, \\& g_{2} ( x ) =\psi^{\prime } \biggl( x+\frac{1}{2} \biggr) -x\frac{x ^{4}+\frac{227}{66}x^{2}+\frac{4237}{2640}}{x^{6}+\frac{155}{44}x^{4}+ \frac{329}{176}x^{2}+\frac{375}{4928}}. \end{aligned}$$

Then we have

$$\begin{aligned} g_{1} ( x+1 ) -g_{1} ( x ) &=\psi^{\prime } \biggl( x+ \frac{3}{2} \biggr) -\frac{1}{x+1}\frac{ ( x+1 ) ^{4}+ \frac{67}{36} ( x+1 ) ^{2}+\frac{256}{945}}{ ( x+1 ) ^{4}+\frac{35}{18} ( x+1 ) ^{2}+\frac{407}{1008}} \\ &\quad {}-\psi^{\prime } \biggl( x+\frac{1}{2} \biggr) + \frac{1}{x}\frac{x^{4}+ \frac{67}{36}x^{2}+\frac{256}{945}}{x^{4}+\frac{35}{18}x^{2}+ \frac{407}{1008}} \\ &=921{,}600\times \bigl[ x ( 2x+1 ) ^{2} ( x+1 ) \bigl( 1008x^{4}+1960x^{2}+407 \bigr) \bigr] ^{-1} \\ &\quad {}\times \bigl( 1008x^{4}+4032x^{3}+8008x^{2}+7952x+3375 \bigr) ^{-1}>0. \end{aligned}$$

Hence, we conclude that

$$ g_{1} ( x ) < g_{1} ( x+1 ) < \cdots < \lim _{n\rightarrow \infty }g_{1} ( x+n ) =0, $$

which proves the first inequality of (2.2).

Analogously, we have

$$\begin{aligned} &g_{2} ( x+1 ) -g_{2} ( x ) \\ &\quad =\psi^{\prime } \biggl( x+ \frac{3}{2} \biggr) - \frac{ ( x+1 ) ( ( x+1 ) ^{4}+\frac{227}{66} ( x+1 ) ^{2}+\frac{4237}{2640} ) }{ ( x+1 ) ^{6}+\frac{155}{44} ( x+1 ) ^{4}+ \frac{329}{176} ( x+1 ) ^{2}+\frac{375}{4928}} \\ &\quad \quad {}-\psi^{\prime } \biggl( x+\frac{1}{2} \biggr) + \frac{x ( x^{4}+ \frac{227}{66}x^{2}+\frac{4237}{2640} ) }{x^{6}+\frac{155}{44}x ^{4}+\frac{329}{176}x^{2}+\frac{375}{4928}} \\ &\quad =-58{,}982{,}400\times \bigl[ ( 2x+1 ) ^{2} \bigl( 4928x^{6}+17{,}360x^{4}+9212x^{2}+375 \bigr) \bigr] ^{-1} \\ &\quad \quad {}\times \bigl( 4928x^{6}+29{,}568x^{5}+91 {,}280x^{4}+168{,}000x^{3} \\ &\quad \quad {}+187{,}292x^{2}+117 {,}432x+31{,}875 \bigr) ^{-1} \\ &\quad < 0. \end{aligned}$$

It then follows that

$$ g_{2} ( x ) >g_{2} ( x+1 ) >\cdots > \lim _{n\rightarrow \infty }g_{2} ( x+n ) =0, $$

which proves the second formula of (2.2). This completes the proof. □

### Lemma 2

*The inequalities*

2.3 $$\begin{aligned}& \frac{t^{2}}{\sinh^{2}t} >1-\frac{1}{3}t^{2}+\frac{1}{15}t^{4}- \frac{2}{189}t^{6} , \end{aligned}$$

2.4 $$\begin{aligned}& \frac{t^{2}}{\sinh^{2}t} >1-\frac{1}{3}t^{2}+\frac{1}{15}t^{4}- \frac{2}{189}t^{6}+\frac{1}{675}t^{8}- \frac{2}{10{,}395}t^{10} \end{aligned}$$

*hold for all* $t>0$.

### Proof

The inequalities in question are equivalent to

$$ h_{1} ( t ) = \biggl( \frac{2}{189}t^{6}- \frac{1}{15}t^{4}+ \frac{1}{3}t^{2}-1 \biggr) \frac{\cosh 2t-1}{2t^{2}}+1>0 $$

and

$$ h_{2} ( t ) = \biggl( \frac{2}{10{,}395}t^{10}- \frac{1}{675}t ^{8}+\frac{2}{189}t^{6}- \frac{1}{15}t^{4}+\frac{1}{3}t^{2}-1 \biggr) \frac{ \cosh 2t-1}{2t^{2}}+1>0 $$

for $t>0$, respectively.

Expanding into a power series yields

$$\begin{aligned} h_{1} ( t ) &= \biggl( \frac{2}{189}t^{6}- \frac{1}{15}t^{4}+ \frac{1}{3}t^{2}-1 \biggr) \sum_{n=0}^{\infty }\frac{2^{2n+1}}{ ( 2n+2 ) !}t^{2n}+1 \\ &=\frac{2}{189}\sum_{n=3}^{\infty } \frac{ ( 2t ) ^{2n}}{32 ( 2n-4 ) !}-\frac{1}{15}\sum_{n=2}^{\infty } \frac{ ( 2t ) ^{2n}}{8 ( 2n-2 ) !}+\frac{1}{3}\sum_{n=1}^{\infty } \frac{ ( 2t ) ^{2n}}{2 ( 2n ) !} \\ &\quad {}-2\sum_{n=0}^{\infty } \frac{ ( 2t ) ^{2n}}{ ( 2n+2 ) !}+1:= \frac{1}{1890}\sum_{n=3}^{\infty } \frac{ ( n-3 ) \times p_{5} ( n ) }{ ( 2n+2 ) !} ( 2t ) ^{2n}, \end{aligned}$$

where

$$ p_{5} ( n ) =40n^{5}+60n^{4}-122n^{3}-543n^{2}-296n+1050. $$

We assert that $p_{5} ( n ) >0$ for $n\geq 3$, since $p_{5} ( n ) $ can be written as

$$ p_{5} ( n ) =40 ( n-3 ) ^{5}+660 ( n-3 ) ^{4}+4198 ( n-3 ) ^{3}+12{,}399 ( n-3 ) ^{2}+15 {,}832 ( n-3 ) +6561, $$

which is evidently positive for $n\geq 3$. Hence $h_{1} ( t ) >0$ for all $t>0$. While

$$\begin{aligned} h_{2} ( t ) &=h_{1} ( t ) + \biggl( \frac{2}{10{,}395}t^{10}- \frac{1}{675}t^{8} \biggr) \sum_{n=0}^{\infty } \frac{2^{2n+1}}{ ( 2n+2 ) !}t^{2n} \\ &=\frac{1}{1890}\sum_{n=3}^{\infty } \frac{ ( n-3 ) \times p _{5} ( n ) }{ ( 2n+2 ) !} ( 2t ) ^{2n}+\frac{2}{10 {,}395}\sum _{n=5}^{\infty }\frac{ ( 2t ) ^{2n}}{2^{9} ( 2n-8 ) !} \\ &\quad {}-\frac{1}{675}\sum_{n=4}^{\infty } \frac{ ( 2t ) ^{2n}}{2^{7} ( 2n-6 ) !}:=\frac{1}{415{,}800}\sum_{n=5}^{\infty } \frac{ ( n-5 ) \times p_{9} ( n ) }{ ( 2n+2 ) !} ( 2t ) ^{2n}, \end{aligned}$$

where

$$\begin{aligned} p_{9} ( n ) &=160n^{9}-1200n^{8}+2368n^{7}-1768n^{6}+2354n ^{5}+14{,}845n^{4} \\ &\quad {}-6403n^{3}-70{,}782n^{2}-57{,}384n+138 {,}600. \end{aligned}$$

It is easy to check that

$$\begin{aligned} p_{9} ( n ) &=160m^{9}+6000m^{8}+98 {,}368m^{7}+921{,}112m^{6}+5392 {,}514m^{5}+20{,}270 {,}695m^{4} \\ &\quad {}+48{,}258{,}997m^{3}+68{,}827 {,}423m^{2}+51{,}883{,}321m+15 {,}041{,}130>0, \end{aligned}$$

for $m=n-5\geq 0$, which proves $h_{2} ( t ) >0$ for $t>0$. The proof is complete. □

The following lemma offers a simple criterion to determine the sign of a class of special polynomials on given interval contained in $( 0,\infty ) $ without using Descartes’ Rule of Signs, which plays an important role in studying certain special functions, see, for example, [39, 40]. A series version can be found in [41, 42].

### Lemma 3

([39, Lemma 7])

*Let* $n\in \mathbb{N}$ *and* $m\in \mathbb{N}\cup \{0\}$ *with* $n>m$ *and let* $P_{n} ( t ) $ *be an* *nth degree polynomial defined by*

2.5 $$ P_{n} ( t ) =\sum_{i=m+1}^{n}a_{i}t^{i}- \sum_{i=0}^{m}a_{i}t ^{i}, $$

*where* $a_{n},a_{m}>0$, $a_{i}\geq 0$ *for* $0\leq i\leq n-1$ *with* $i\neq m$. *Then there is a unique number* $t_{m+1}\in ( 0,\infty ) $ *satisfying* $P_{n} (t_{m+1}) =0$ *such that* $P_{n} ( t ) <0$ *for* $t\in ( 0,t_{m+1} ) $ *and* $P_{n} ( t ) >0$ *for* $t\in ( t_{m+1},\infty ) $.

*Consequently*, *for a given* $t_{0}>0$, *if* $P_{n} ( t_{0} ) >0$ *then* $P_{n} ( t ) >0$ *for* $t\in ( t_{0},\infty ) $ *and if* $P_{n} ( t_{0} ) <0$ *then* $P_{n} ( t ) <0$ *for* $t\in ( 0,t_{0} ) $.

## Monotonicity and convexity

### Theorem 1

*T he function*

$$ f_{0} ( x ) =\ln \Gamma ( x+1 ) -\ln \sqrt{2 \pi }- \biggl( x+ \frac{1}{2} \biggr) \ln x+x-\frac{x}{2}\ln \biggl( x \sinh \frac{1}{x} \biggr) $$

*is strictly decreasing and convex on* $( 0,\infty ) $.

### Proof

Differentiation yields

$$\begin{aligned}& f_{0}^{\prime } ( x ) = \psi ( x+1 ) -\frac{1}{2} \ln \biggl( x\sinh \frac{1}{x} \biggr) +\frac{1}{2x}\coth \frac{1}{x}- \ln x-\frac{1}{2x}-\frac{1}{2}, \\& f_{0}^{\prime \prime } ( x ) = \psi ' ( x+1 ) + \frac{1}{2x ^{3}}\frac{1}{\sinh^{2} ( 1/x ) }-\frac{3}{2x}+\frac{1}{2x ^{2}}. \end{aligned}$$

Replacing *x* by $( x+1/2 ) $ in inequality (2.1) leads to

$$ \psi^{\prime } ( x+1 ) >\frac{5}{6}\frac{ ( 2x+1 ) ( 21x^{2}+21x+23 ) }{35x^{4}+70x^{3}+85x^{2}+50x+12}, $$

and using which to $f_{0}^{\prime \prime } ( x ) $ gives

$$\begin{aligned} f_{0}^{\prime \prime } ( x ) &>\frac{5}{6}\frac{ ( 2x+1 ) ( 21x^{2}+21x+23 ) }{35x^{4}+70x^{3}+85x^{2}+50x+12} \\ &\quad {}+\frac{1}{2x^{3}}\frac{1}{\sinh^{2} ( 1/x ) }- \frac{3}{2x}+ \frac{1}{2x^{2}}=f_{01} \biggl( \frac{1}{x} \biggr) . \end{aligned}$$

Simplifying yields

$$\begin{aligned} f_{01} ( t ) &=\frac{1}{2}\frac{t^{3}}{\sinh^{2}t}+ \frac{1}{6}\frac{t ( 36t^{5}+42t^{4}-80t^{3}-220t^{2}-210t-105 ) }{12t^{4}+50t^{3}+85t ^{2}+70t+35} \\ &=\frac{t}{12}\frac{f_{02} ( t ) }{ ( 12t^{4}+50t^{3}+85t ^{2}+70t+35 ) \sinh^{2}t}, \end{aligned}$$

where

$$\begin{aligned} f_{02} ( t ) &= \bigl( 36t^{5}+42t^{4}-80t^{3}-220t^{2}-210t-105 \bigr) \cosh 2t \\ &\quad {}+ \bigl( 72t^{6}+264t^{5}+468t^{4}+500t^{3}+430t^{2}+210t+105 \bigr) . \end{aligned}$$

Expanding into a power series gives

$$\begin{aligned} f_{02} ( t ) &= \Biggl( 36\sum_{n=2}^{\infty } \frac{2^{2n-4}}{ ( 2n-4 ) !}t^{2n+1}-80\sum_{n=1}^{\infty } \frac{2^{2n-2}}{ ( 2n-2 ) !}t^{2n+1}-210\sum_{n=0}^{\infty } \frac{2^{2n}}{ ( 2n ) !}t^{2n+1} \Biggr) \\ &\quad {}+ \Biggl( 42\sum_{n=2}^{\infty } \frac{2^{2n-4}}{ ( 2n-4 ) !} t^{2n}-220\sum_{n=1}^{\infty } \frac{2^{2n-2}}{ ( 2n-2 ) !} t ^{2n}-105\sum_{n=0}^{\infty } \frac{2^{2n}}{ ( 2n ) !}t^{2n} \Biggr) \\ &\quad {}+ \bigl( 72t^{6}+264t^{5}+468t^{4}+500t^{3}+430t^{2}+210t+105 \bigr) \\ &=\sum_{n=4}^{\infty }\frac{ ( n-3 ) ( 36n^{3}+19n+70 ) 2^{2n}}{ ( 2n ) !}t^{2n+1} \\ &\quad {}+\sum_{n=4}^{\infty }\frac{ ( 84n^{4}-252n^{3}-209n^{2}+157n-210 ) 2^{2n-2}}{ ( 2n ) !}t ^{2n}>0, \end{aligned}$$

where the inequality holds due to

$$\begin{aligned} &84n^{4}-252n^{3}-209n^{2}+157n-210 \\ &\quad =84 ( n-4 ) ^{4}+1092 ( n-4 ) ^{3}+4831 ( n-4 ) ^{2}+7893 ( n-4 ) +2450>0 \end{aligned}$$

for $n\geq 4$.

It then follows that $f_{01} ( t ) >0$ for $t>0$, so $f_{0}^{\prime \prime } ( x ) >0$ for $x>0$. This yields $f_{0}^{\prime } ( x ) <\lim_{x\rightarrow \infty }f_{0}^{ \prime } ( x ) =0$, which proves the desired result. □

### Theorem 2

*The function*

$$ f_{1}^{\ast } ( x ) =\ln \Gamma ( x+1 ) -\ln \sqrt{2 \pi }- \biggl( x+\frac{1}{2} \biggr) \ln x+x-\frac{x}{2}\ln \biggl( x \sinh \frac{1}{x} \biggr) -\ln \biggl( 1+\frac{1}{1620x^{5}} \biggr) $$

*is strictly increasing and concave on* $( 0,\infty ) $.

### Proof

Differentiation yields

$$\begin{aligned} f_{1}^{\ast \prime } ( x ) &=\psi ( x+1 ) - \frac{1}{2}\ln \biggl( x\sinh \frac{1}{x} \biggr) +\frac{1}{2x}\coth \frac{1}{x} \\ &\quad {}-\ln x-\frac{1}{2x}-\frac{1}{2}+\frac{5}{x ( 1620x^{5}+1 ) }, \\ f_{1}^{\ast \prime \prime } ( x ) &=\psi^{\prime } ( x+1 ) + \frac{1}{2x^{3}}\frac{1}{\sinh^{2} ( 1/x ) }- \frac{3}{2x}+\frac{1}{2x^{2}}-5 \frac{9720x^{5}+1}{x^{2} ( 1620x ^{5}+1 ) ^{2}}. \end{aligned}$$

Since $\lim_{x\rightarrow \infty }f_{1}^{\ast \prime } ( x ) =0$, it suffices to prove $f_{1}^{\ast \prime \prime } ( x ) <0$ for $x>0 $. Replacing *x* by $( x+1/2 ) $ in the right-hand side inequality of (2.2) leads to

3.1 $$ \psi^{\prime } ( x+1 ) < \frac{1}{30}\frac{3780x^{4}+7560x ^{3}+12{,}705x^{2}+8925x+3019}{ ( 2x+1 ) ( 63x^{4}+126x ^{3}+217x^{2}+154x+60 ) }, $$

which indicates that

$$\begin{aligned} f_{1}^{\ast \prime \prime } ( x ) &< \frac{1}{30}\frac{3780x ^{4}+7560x^{3}+12{,}705x^{2}+8925x+3019}{ ( 2x+1 ) ( 63x ^{4}+126x^{3}+217x^{2}+154x+60 ) } \\ &\quad {}+\frac{1}{2x^{3}}\frac{1}{\sinh^{2} ( 1/x ) }- \frac{3}{2x}+ \frac{1}{2x^{2}}-5\frac{9720x^{5}+1}{x^{2} ( 1620x ^{5}+1 ) ^{2}}:=f_{11}^{\ast } \biggl( \frac{1}{x} \biggr) , \end{aligned}$$

where

$$\begin{aligned} f_{11}^{\ast } ( t ) &=\frac{t^{3}}{\cosh 2t-1}-\frac{3}{2}t+ \frac{1}{2}t^{2}-5t^{7}\frac{t^{5}+9720}{ ( t^{5}+1620 ) ^{2}} \\ &\quad {}+\frac{1}{30}\frac{t ( 3019t^{4}+8925t^{3}+12{,}705t^{2}+7560t+3780 ) }{ ( t+2 ) ( 60t^{4}+154t^{3}+217t^{2}+126t+63 ) }. \end{aligned}$$

Using the inequality

$$ \cosh 2t-1>\sum_{n=1}^{4} \frac{2^{2n}}{ ( 2n ) !}t^{2n}=2t ^{2}+\frac{2}{3}t^{4}+ \frac{4}{45}t^{6}+\frac{2}{315}t^{8} $$

yields

$$\begin{aligned} &f_{11}^{\ast } ( t ) \\ &\quad < \frac{t^{3}}{2t^{2}+\frac{2}{3}t^{4}+ \frac{4}{45}t^{6}+\frac{2}{315}t^{8}}-\frac{3}{2}t+ \frac{1}{2}t^{2}-5t ^{7}\frac{t^{5}+9720}{ ( t^{5}+1620 ) ^{2}} \\ &\quad \quad {}+\frac{1}{30}\frac{t ( 3019t^{4}+8925t^{3}+12{,}705t^{2}+7560t+3780 ) }{ ( t+2 ) ( 60t^{4}+154t^{3}+217t^{2}+126t+63 ) } \\ &\quad =-\frac{1}{30} \\ &\quad\quad {}\times\frac{t^{9}\times f_{12}^{\ast } ( t ) }{ ( t^{5}+1620 ) ^{2} ( t+2 ) ( t^{6}+14t^{4}+105t^{2}+315 ) ( 60t^{4}+154t^{3}+217t^{2}+126t+63 ) } \\ &\quad < 0 \end{aligned}$$

for $t>0$, where the inequality holds due to

$$\begin{aligned} f_{12}^{\ast } ( t ) &=8100t^{14}+39{,}690t^{13}+193{,}586t ^{12}+645{,}960t^{11}+2{,}028{,}124t^{10} \\ &\quad {}+90{,}019{,}275t^{9}+406{,}666 {,}800t^{8}+1976{,}029 {,}740t^{7}+6395 {,}589{,}900t^{6} \\ &\quad {}+20{,}173{,}546{,}260t^{5}+51{,}035 {,}406{,}750t^{4}+110{,}592 {,}337{,}500t ^{3} \\ &\quad {}+184{,}843{,}490{,}400t^{2}+254{,}068 {,}164{,}000t+101{,}627{,}265{,}600>0 \end{aligned}$$

for $t>0$. This implies that $f_{2}^{\prime \prime } ( x ) <0$ for all $x>0$, and the proof is complete. □

### Theorem 3

*The function*

$$ f_{1} ( x ) =\ln \Gamma ( x+1 ) -\ln \sqrt{2 \pi }- \biggl( x+ \frac{1}{2} \biggr) \ln x+x-\frac{x}{2}\ln \biggl( x \sinh \frac{1}{x} \biggr) -\frac{1}{1620x^{5}} $$

*is strictly increasing and concave on* $( 0,\infty ) $.

### Proof

We clearly see that

$$ f_{1} ( x ) =f_{1}^{\ast } ( x ) +D \biggl( \frac{1}{1620x ^{5}} \biggr) , $$

where $D ( y ) =\ln ( 1+y ) -y$. By Theorem 2, $f_{1}^{\ast }$ is strictly increasing and concave on $( 0,\infty ) $, so if we prove $x\mapsto D ( y ) $ is strictly increasing and concave on $( 0,\infty ) $, then so will be $f_{1}$, and the proof will be complete. Now we easily check that for $x>0$,

$$\begin{aligned}& \frac{dD ( y ) }{dx} =\frac{1}{324x^{6} (1620x^{5}+1 ) }>0, \\& \frac{d^{2}D ( y ) }{dx^{2}} =-\frac{1}{54}\frac{2970x^{5}+1}{x ^{7} ( 1620x^{5}+1 ) ^{2}}< 0, \end{aligned}$$

which completes the proof. □

### Theorem 4

*The function*

$$\begin{aligned} f_{2} ( x ) &=\ln \Gamma ( x+1 ) -\ln \sqrt{2 \pi }- \biggl( x+ \frac{1}{2} \biggr) \ln x+x \\ &\quad {}-\frac{x}{2}\ln \biggl( x\sinh \frac{1}{x} \biggr) - \frac{1}{1620x ^{5}}+\frac{11}{18{,}900x^{7}} \end{aligned}$$

*is strictly decreasing and convex on* $( 0,\infty ) $.

### Proof

Differentiation yields

$$\begin{aligned}& f_{2}^{\prime } ( x ) =\psi ( x+1 ) -\frac{1}{2} \ln \biggl( x\sinh \frac{1}{x} \biggr) +\frac{1}{2x}\coth \frac{1}{x} \\& \hphantom{f_{2}^{\prime } ( x ) =}{}-\ln x-\frac{1}{2x}-\frac{1}{2}+ \frac{1}{324x^{6}}- \frac{11}{2700x ^{8}}, \\& f_{2}^{\prime \prime } ( x )=\psi^{\prime } ( x+1 ) + \frac{1}{2x^{3}}\frac{1}{\sinh^{2} ( 1/x ) }-\frac{3}{2x}+\frac{1}{2x ^{2}}- \frac{1}{54x^{7}}+\frac{22}{675x^{9}}. \end{aligned}$$

Since $\lim_{x\rightarrow \infty }f_{2}^{\prime } ( x ) =0$, it suffices to prove $f_{2}^{\prime \prime } ( x ) >0$ for $x>0$. Replacing *x* by $( x+1/2 ) $ in the left-hand side inequality of (2.2) leads to

$$ \psi^{\prime } ( x+1 ) >\frac{7}{30}\frac{ ( 2x+1 ) ( 165x^{4}+330x^{3}+815x^{2}+650x+417 ) }{77x^{6}+231x^{5}+560x ^{4}+735x^{3}+623x^{2}+294x+60}, $$

and applying which to $f_{2}^{\prime \prime } ( x ) $ gives

$$\begin{aligned} f_{2}^{\prime \prime } ( x ) &>\frac{7}{30}\frac{ ( 2x+1 ) ( 165x^{4}+330x^{3}+815x^{2}+650x+417 ) }{ 77x ^{6}+231x^{5}+560x^{4}+735x^{3}+623x^{2}+294x+60} \\ &\quad {}+\frac{1}{2x^{3}}\frac{1}{\sinh^{2} ( 1/x ) }-\frac{3}{2x}+ \frac{ 1}{2x^{2}}- \frac{1}{54x^{7}}+ \frac{22}{675x^{9}}=f_{21} \biggl( \frac{1}{x} \biggr) . \end{aligned}$$

Making a change of variable $t=1/x$ yields

$$\begin{aligned} f_{21} ( t ) &=\frac{7}{30}\frac{t ( t+2 ) ( 417t ^{4}+650t^{3}+815t^{2}+330t+165 ) }{60t^{6}+294t^{5}+623t^{4}+735t ^{3}+560t^{2}+231t+77} \\ &\quad {}-\frac{3}{2}t+\frac{1}{2}t^{2}- \frac{1}{54}t^{7}+ \frac{22}{675}t ^{9}+ \frac{t}{2}\frac{t^{2}}{\sinh^{2}t}. \end{aligned}$$

We distinguish two cases to prove $f_{21} ( t ) >0$ for all $t>0$.

*Case* 1: $t\geq 1$. Application of inequality (2.3) gives

$$\begin{aligned} f_{21} ( t ) &>\frac{7}{30}\frac{t ( t+2 ) ( 417t ^{4}+650t^{3}+815t^{2}+330t+165 ) }{60t^{6}+294t^{5}+623t^{4}+735t ^{3}+560t^{2}+231t+77}- \frac{3}{2}t+\frac{1}{2}t^{2} \\ &\quad {}-\frac{1}{54}t^{7}+\frac{22}{675}t^{9}+ \frac{t}{2} \biggl( 1- \frac{1}{3}t^{2}+ \frac{1}{15}t^{4}-\frac{2}{189}t^{6} \biggr) \end{aligned}$$

$$ =\frac{1}{9450}\frac{t^{9}\times p_{6} ( t ) }{60t^{6}+294t ^{5}+623t^{4}+735t^{3}+560t^{2}+231t+77}, $$

where

$$ p_{6} ( t ) =18{,}480t^{6}+90{,}552t^{5}+178 {,}384t^{4}+160{,}230t ^{3}+51 {,}205t^{2}-1617t-539. $$

Clearly, $p_{6} ( t ) >0$ for $t\geq 1$, so $f_{21} ( t ) >0$ for $t\geq 1$.

*Case* 2: $0< t<1$. Using inequality (2.4) yields

$$\begin{aligned} f_{21} ( t ) &>\frac{7}{30}\frac{t ( t+2 ) ( 417t ^{4}+650t^{3}+815t^{2}+330t+165 ) }{60t^{6}+294t^{5}+623t^{4}+735t ^{3}+560t^{2}+231t+77}- \frac{3}{2}t+\frac{1}{2}t^{2} \\ &\quad {}-\frac{1}{54}t^{7}+\frac{22}{675}t^{9}+ \frac{t}{2} \biggl( 1- \frac{1}{3}t^{2}+ \frac{1}{15}t^{4}-\frac{2}{189}t^{6}+ \frac{1}{675}t ^{8}-\frac{2}{10{,}395}t^{10} \biggr) \end{aligned}$$

$$ =\frac{t^{11}\times q_{6} ( t ) }{60t^{6}+294t^{5}+623t^{4}+735t ^{3}+560t^{2}+231t+77}, $$

where

$$ q_{6} ( t ) =-\frac{4}{693}t^{6}- \frac{14}{495}t^{5}+ \frac{2881}{1485}t^{4}+ \frac{4816}{495}t^{3}+\frac{400{,}919}{20{,}790}t ^{2}+ \frac{1573}{90}t+\frac{1573}{270}. $$

Since the coefficients of polynomial $q_{6} ( t ) $ satisfy the conditions of Lemma 3 and $q_{6} ( 1 ) =53{,}681/990>0$, we find that $q_{6} ( t ) >0$ for $t\in ( 0,1 ) $, and then $f_{21} ( t ) >0$ for $t\in ( 0,1 ) $.

This ends the proof. □

### Theorem 5

*The function*

$$\begin{aligned} f_{2}^{\ast } ( x ) &=\ln \Gamma ( x+1 ) -\ln \sqrt{2 \pi }- \biggl( x+\frac{1}{2} \biggr) \ln x+x \\ &\quad {}-\frac{x}{2}\ln \biggl( x\sinh \frac{1}{x} \biggr) -\ln \biggl( 1+\frac{1}{1620x ^{5}}-\frac{11}{18{,}900x^{7}} \biggr) \end{aligned}$$

*is strictly decreasing and convex on* $[4/3,\infty )$.

### Proof

We easily see that

$$ f_{2}^{\ast } ( x ) =f_{2} ( x ) -D \biggl( \frac{1}{1620x ^{5}}-\frac{11}{18{,}900x^{7}} \biggr) , $$

where $D ( y ) =\ln ( 1+y ) -y$. By Theorem 4, $f_{2}$ is strictly decreasing and convex on $( 0, \infty ) $, so if we prove $x\mapsto D ( y ) $ is strictly increasing and concave on $[4/3,\infty )$, then so will be $f_{2}^{\ast }$, and the proof will be complete. Now we easily check that for $x\geq 4/3$,

$$\begin{aligned}& \frac{dD ( y ) }{dx} = \frac{1}{8100}\frac{ ( 25x^{2}-33 ) ( 35x^{2}-33 ) }{x^{8} ( 35x^{2}+56{,}700x ^{7}-33 ) }>0, \\& \frac{d^{2}D ( y ) }{dx^{2}} = -\frac{p_{11} ( x ) }{x^{9} ( 56{,}700x^{7}+35x^{2}-33 ) ^{2}}< 0, \end{aligned}$$

where the last inequality holds due to

$$\begin{aligned} p_{11} ( x ) &=67{,}375x^{11}-180{,}180x^{9}+114 {,}345x^{7}+ \frac{1225}{54}x^{6}- \frac{2233}{27}x^{4}+ \frac{8591}{90}x^{2}- \frac{2662}{75} \\ &=385x^{7} \biggl[ \biggl( 175 \biggl( x^{2}- \frac{16}{9} \biggr) ^{2}+ \frac{1388}{9} \biggl( x^{2}-\frac{16}{9} \biggr) +\frac{1465}{81} \biggr) \biggr] \\ &\quad {}+\frac{1}{1350}x^{2} \bigl( 175x^{2}-319 \bigr) ^{2}+\frac{242}{675} \biggl[ 56 \biggl( x^{2}- \frac{16}{9} \biggr) +\frac{5}{9} \biggr] >0, \end{aligned}$$

which completes the proof. □

## Inequalities

As is well known, analytic inequalities [43–45] play a very important role in different branches of modern mathematics. Using the theorems presented in the previous section, we can obtain some new inequalities for the gamma function and factorial function related to Windschitl’s formula.

### Corollary 1

*Let* $W_{0} ( x ) $ *be defined by* (1.2). *Then the inequalities*

4.1 $$ \max \biggl( 1,\exp \biggl( \frac{1}{1620x^{5}}-\frac{11}{18{,}900x ^{7}} \biggr) \biggr) < \frac{\Gamma ( x+1 ) }{W_{0} ( x ) }< 1+\frac{1}{1620x^{5}}< \exp \biggl( \frac{1}{1620x^{5}} \biggr) $$

*hold for all* $x>0$. *If* $x\geq \sqrt{33/35}$, *then we have*

4.2 $$\begin{aligned} 1+\frac{1}{1620x^{5}}-\frac{11}{18{,}900x^{7}}&< \exp \biggl( \frac{1}{1620x ^{5}}- \frac{11}{18{,}900x^{7}} \biggr) \\ &< \frac{\Gamma ( x+1 ) }{W_{0} ( x ) }< 1+\frac{1}{1620x ^{5}}< \exp \biggl( \frac{1}{1620x^{5}} \biggr). \end{aligned}$$

### Proof

The first and second inequalities in (4.1) follow directly from the monotonicity of $f_{0}$, $f_{2}$ and $f_{1}^{\ast }$ on $( 0,\infty ) $ given in Theorems 1, 4 and 2, respectively, due to $f_{0} ( \infty ) =$ $f_{2} ( \infty ) =f_{1}^{\ast } ( \infty ) =0$. The third one holds due to a simple inequality $1+y< e^{y}$ for $y>0$. The proof of inequalities (4.2) is similar, which completes the proof. □

Using the monotonicity of $f_{0}$, $f_{1}^{\ast }$ and $f_{2}$ on $( 0,\infty ) $ and noting that

$$ f_{0} ( 1 ) =\ln \frac{e}{\sqrt{2\pi \sinh 1}}, \qquad f _{1}^{\ast } ( 1 ) =\ln \biggl( \frac{1620}{1621}\frac{e}{\sqrt{2 \pi \sinh 1}} \biggr),\qquad f_{2} ( 1 ) =\ln \frac{e^{28{,}349/28 {,}350}}{\sqrt{2\pi \sinh 1}}, $$

we immediately get the following corollary.

### Corollary 2

*For* $n\in \mathbb{N}$, *the inequalities*

$$\begin{aligned}& 1 < \frac{n!}{\sqrt{2\pi n} ( \frac{n}{e} ) ^{n} ( n \sinh \frac{1}{n} ) ^{n/2}}\leq \alpha_{0}, \\& \beta_{1}^{\ast } \biggl( 1+\frac{1}{1620n^{5}} \biggr) \leq \frac{n!}{\sqrt{2 \pi n} ( \frac{n}{e} ) ^{n} ( n\sinh \frac{1}{n} ) ^{n/2}}< 1+\frac{1}{1620n^{5}}, \end{aligned}$$

$$ \exp \biggl( \frac{1}{1620n^{5}}-\frac{11}{18{,}900n^{7}} \biggr) < \frac{n!}{\sqrt{2 \pi n} ( \frac{n}{e} ) ^{n} ( n\sinh \frac{1}{n} ) ^{n/2}}\leq \alpha_{2}\exp \biggl( \frac{1}{1620n^{5}}- \frac{11}{18 {,}900n^{7}} \biggr) $$

*hold with the best constants* $\alpha_{0}=e/\sqrt{2\pi \sinh 1} \approx 1.000{,}34$, $\beta_{1}^{\ast }=1620e/ ( 1621\sqrt{2 \pi \sinh 1} ) \approx 0.999{,}72$ *and* $\alpha_{2}=e^{28{,}349/28 {,}350}/\sqrt{2\pi \sinh 1}\approx 1.000{,}30$.

The proof of inequalities (1.4) presented by Alzer [37] seems to be somewhat complicated. With the aid of the first and second inequalities in (4.1), we can give a new and simpler proof.

### Proof of inequalities (1.4)

The sufficiency for the inequalities (1.4) to hold for $x>0$ follows by the first and second inequalities in (4.1). The necessary condition for the left-hand side inequality of (1.4) to hold for $x>0$ follows from the following relation:

$$\begin{aligned} &\lim_{x\rightarrow 0^{+}}\frac{\ln \Gamma ( x+1 ) - \ln \sqrt{2\pi }- ( x+\frac{1}{2} ) \ln x+x-\frac{x}{2} \ln ( x\sinh \frac{1}{x} ) -\ln ( 1+\frac{\alpha }{x ^{5}} ) }{\ln ( 1/x ) } \\ &\quad = \textstyle\begin{cases} \frac{1}{2} &\text{if }\alpha =0, \\ -\frac{9}{2} &\text{if }\alpha \neq 0. \end{cases}\displaystyle \end{aligned}$$

While the necessary condition for the right-hand side of (1.4) to hold for $x>0$ follows from the limit relation

$$\begin{aligned}& \lim_{x\rightarrow \infty }\frac{\ln \Gamma ( x+1 ) - \ln \sqrt{2\pi }- ( x+\frac{1}{2} ) \ln x+x-\frac{x}{2} \ln ( x\sinh \frac{1}{x} ) -\ln ( 1+\frac{\beta }{x ^{5}} ) }{x^{-5}} \\& \quad =\frac{1}{1620}-\beta \leq 0. \end{aligned}$$

This completes the proof. □

The following corollary offers a strengthening for Lu et al.’s inequalities (1.6).

### Corollary 3

*The inequalities*

4.3 $$\begin{aligned} \biggl[ x\sinh \biggl( \frac{1}{x}+\frac{1}{810x^{7}}-\frac{67}{42{,}525x ^{9}} \biggr) \biggr] ^{x/2} &< \biggl( x\sinh \frac{1}{x} \biggr) ^{x/2}\exp \biggl( \frac{1}{1620x^{5}}-\frac{11}{18{,}900x^{7}} \biggr) \\ &< \frac{\Gamma ( x+1 ) }{\sqrt{2\pi x} ( x/e )^{x}} \\ &< \biggl( x\sinh \frac{1}{x} \biggr) ^{x/2}\exp \biggl( \frac{1}{1620x ^{5}} \biggr) \\ & < \biggl[ x\sinh \biggl( \frac{1}{x}+\frac{1}{810x^{7}} \biggr) \biggr] ^{x/2} \end{aligned}$$

*hold for* $x>c$, *where* $c=0$ *for the second*, *third and fourth inequalities*, *while* $c=x_{0}\approx 0.43738$ *for the first one*, *here* $x_{0}$ *is the unique solution of the equation*

$$ \frac{1}{x}+\frac{1}{810x^{7}}-\frac{67}{42{,}525x^{9}}=0 $$

*on* $( 0,\infty ) $.

### Proof

Clearly, the second and third inequalities of (4.3) follow by the first and second inequalities in (4.1). It remains to prove the first and last inequalities of (4.3).

(i) The last one is equivalent to

$$ \frac{x}{2}\ln \biggl[ x\sinh \biggl( \frac{1}{x}+ \frac{1}{810x^{7}} \biggr) \biggr] >\frac{x}{2}\ln \biggl( x \sinh \frac{1}{x} \biggr) +\frac{1}{1620x^{5}}, $$

or equivalently,

$$ h_{3} ( t ) =\ln \sinh \biggl( t+\frac{1}{810}t^{7} \biggr) - \ln \sinh t-\frac{1}{810}t^{6}>0 $$

for $t=1/x>0$. Denote by $l ( t ) =\ln \sinh t$ and $t_{2}= ( t+t^{7}/810 ) $. Then by Taylor formula we have

$$ h_{3} ( t ) =l ( t_{2} ) -l ( t ) - \frac{1}{810}t^{6}= ( t_{2}-t ) l^{\prime } ( t ) +\frac{l ^{\prime \prime } ( t ) }{2!} ( t_{2}-t ) ^{2}+\frac{l ^{\prime \prime \prime } ( \xi ) }{3!} ( t_{2}-t ) ^{3}-\frac{1}{810}t^{6}, $$

where $t<\xi <t+t^{7}/810$. Since $l^{\prime \prime \prime } ( t ) =2 ( \cosh t ) /\sinh^{3}t>0$, we get

$$ h_{3} ( t ) >\frac{1}{810}t^{7}\frac{\cosh t}{\sinh t}- \frac{t ^{14}}{2\times 810^{2}}\frac{1}{\sinh^{2}t}-\frac{1}{810}t^{6}:= \frac{t ^{6}\times h_{31} ( t ) }{2\times 810^{2}\sinh^{2}t}, $$

where

$$ h_{31} ( t ) =810t\sinh 2t-810\cosh 2t+810-t^{8}. $$

Due to

$$ h_{31} ( t ) =540t^{4}+144t^{6}+ \frac{101}{7}t^{8}+810\sum_{n=5}^{\infty } \frac{ ( n-1 ) 2^{2n}}{ ( 2n ) !}t ^{2n}>0, $$

we conclude that $h_{3} ( t ) >0$ for $t>0$.

(ii) To ensure that the first inequality holds, it is necessary to establish

$$ x\sinh \biggl( \frac{1}{x}+\frac{1}{810x^{7}}-\frac{67}{42{,}525x^{9}} \biggr) >0 $$

for $x>0$, for which it suffices so show that

$$ \frac{1}{x}+\frac{1}{810x^{7}}-\frac{67}{42{,}525x^{9}}=\frac{85{,}050x ^{8}+105x^{2}-134}{85{,}050x^{9}}>0. $$

By Lemma 3, the numerator in the above fraction, as an 8th degree polynomial, has a unique zero $x_{0}$ on $( 0,\infty ) $. Numeric computation gives $x_{0}\approx 0.437{,}38$.

Now the first inequality is equivalent to

$$ \frac{x}{2}\ln \biggl[ x\sinh \biggl( \frac{1}{x}+ \frac{1}{810x^{7}}-\frac{67}{42{,}525x ^{9}} \biggr) \biggr] < \frac{x}{2}\ln \biggl( x\sinh \frac{1}{x} \biggr) +\frac{1}{1620x^{5}}-\frac{11}{18{,}900x^{7}}, $$

or equivalently,

$$ h_{4} ( t ) =\ln \biggl[ \sinh \biggl( t+\frac{1}{810}t^{7}- \frac{67}{42{,}525}t^{9} \biggr) \biggr] -\ln ( \sinh t ) - \frac{1}{810}t^{6}+\frac{11}{9450}t^{8}< 0 $$

for $t=1/x\in ( 0,1/x_{0} ) $, where $1/x_{0}\approx 2.28632$ is clearly the unique zero of the polynomial

$$ t_{1}\equiv t_{1} ( t ) =t+\frac{1}{810}t^{7}- \frac{67}{42{,}525}t^{9} $$

on $( 0,\infty ) $. In view of $l^{\prime \prime } ( t ) =-1/\sinh^{2}t<0$, we have

$$\begin{aligned} h_{4} ( t ) &=l ( t_{1} ) -l ( t ) - \frac{1}{810}t^{6}+ \frac{11}{9450}t^{8}< ( t_{1}-t ) l^{ \prime } ( t ) -\frac{1}{810}t^{6}+ \frac{11}{9450}t^{8} \\ &= \biggl( \frac{1}{810}t^{7}-\frac{67}{42{,}525}t^{9} \biggr) \frac{ \cosh t}{\sinh t}-\frac{1}{810}t^{6}+ \frac{11}{9450}t^{8} \\ &=-\frac{1}{85{,}050}\frac{t^{6}}{\sinh t} \bigl( 105\sinh t-105t \cosh t+134t^{3}\cosh t-99t^{2}\sinh t \bigr) \\ &=-\frac{1}{85{,}050}\frac{t^{6}}{\sinh t}\sum_{n=3}^{\infty } \frac{4 ( 268n-99 ) ( n-1 ) ( n-2 ) }{ ( 2n-1 ) !}t^{2n-1}< 0, \end{aligned}$$

which completes the proof. □

### Remark 1

Clearly, the proof of Corollary 3 can also be regarded as a new proof of Lu et al.’s inequalities (1.6). Moreover, our proof gives the minimum value of *m*, i.e., $\min ( m ) =x_{0} \approx 0.437{,}38$, such that the the double inequality (1.6) holds for all $x>x_{0}$.

## Numeric comparisons

By the asymptotic expansion listed in [46, Eq. (6.1.40)]

5.1 $$ \ln \Gamma ( x+1 ) \thicksim \ln \sqrt{2\pi }+ \biggl( x+ \frac{1}{2} \biggr) \ln x-x+\sum_{n=1}^{\infty } \frac{B_{2n}}{2n ( 2n-1 ) x^{2n-1}}, $$

we easily verify that our four approximation formulas $W_{01} ( x ) $, $W_{01}^{\ast } ( x ) $, $W_{02} ( x ) $ and $W_{02}^{\ast } ( x ) $, defined by (1.8), (1.10), (1.9) and (1.11), respectively, have the following limit relations:

$$\begin{aligned}& \lim_{x\rightarrow \infty }\frac{\ln \Gamma ( x+1 ) -\ln W _{01} ( x ) }{x^{-7}} = \lim_{x\rightarrow \infty } \frac{ \ln \Gamma ( x+1 ) -\ln W_{01}^{\ast } ( x ) }{x ^{-7}}=-\frac{198}{340{,}200}, \\& \lim_{x\rightarrow \infty }\frac{\ln \Gamma ( x+1 ) -\ln W _{02} ( x ) }{x^{-9}} = \lim_{x\rightarrow \infty } \frac{ \ln \Gamma ( x+1 ) -\ln W_{02}^{\ast } ( x ) }{x ^{-9}}=\frac{143}{170{,}100}. \end{aligned}$$

Also, for another approximation formula $W_{1} ( x ) $ defined by (1.3), we have

$$ \lim_{x\rightarrow \infty }\frac{\ln \Gamma ( x+1 ) -\ln W _{1} ( x ) }{x^{-7}}=-\frac{163}{340{,}200}. $$

Denote the two approximation formulas generated by the double inequality (1.6) by

5.2 $$\begin{aligned}& W_{L1} ( x ) = \sqrt{2\pi x} \biggl( \frac{x}{e} \biggr) ^{x} \biggl[ x\sinh \biggl( \frac{1}{x}+\frac{1}{810x^{7}} \biggr) \biggr] ^{x/2}, \end{aligned}$$

5.3 $$\begin{aligned}& W_{L2} ( x ) = \sqrt{2\pi x} \biggl( \frac{x}{e} \biggr) ^{x} \biggl[ x\sinh \biggl( \frac{1}{x}+\frac{1}{810x^{7}}- \frac{67}{42{,}525x ^{9}} \biggr) \biggr] ^{x/2}. \end{aligned}$$

We have

$$\begin{aligned}& \lim_{x\rightarrow \infty }\frac{\ln \Gamma ( x+1 ) -\ln W _{L1} ( x ) }{x^{-7}} = -\frac{67}{85{,}050}, \\& \lim_{x\rightarrow \infty }\frac{\ln \Gamma ( x+1 ) -\ln W _{L2} ( x ) }{x^{-9}} = \frac{19}{17{,}010}. \end{aligned}$$

These, in combination with Corollaries 2, 1 and 3, show that the approximation formula $W_{02} ( x ) $ given by (1.9) is the best among those listed above, which can be seen from comparison Table 1.

**Table 1:** Comparison among $W_{02}$ (1.9), $W_{L2}$ (5.3), $W_{1}$ (1.3), $W_{01}^{\ast }$ (1.10) and $W_{L1}$ (5.2)

*n*	$\vert \frac{W_{02} ( n ) -n!}{n!} \vert $	$\vert \frac{W_{L2} ( n ) -n!}{n!} \vert $	$\vert \frac{W_{1} ( x ) -n!}{n!} \vert $	$\vert \frac{W_{01}^{\ast } ( x ) -n!}{n!} \vert $	$\vert \frac{W_{L1} ( x ) -n!}{n!} \vert $
1	3.065 × 10^−4^	5.655 × 10^−4^	1.832 × 10^−4^	2.754 × 10^−4^	4.686 × 10^−4^
2	1.098 × 10^−6^	1.629 × 10^−6^	2.668 × 10^−6^	3.449 × 10^−6^	5.030 × 10^−6^
5	3.956 × 10^−10^	5.367 × 10^−10^	5.743 × 10^−9^	7.054 × 10^−9^	9.681 × 10^−9^
10	8.221 × 10^−13^	1.098 × 10^−12^	4.710 × 10^−11^	5.738 × 10^−11^	7.794 × 10^−11^
20	1.630 × 10^−15^	2.172 × 10^−15^	3.727 × 10^−13^	4.531 × 10^−13^	6.138 × 10^−13^
50	4.300 × 10^−19^	5.715 × 10^−19^	6.129 × 10^−16^	7.445 × 10^−16^	1.008 × 10^−15^
100	8.404 × 10^−22^	1.117 × 10^−21^	4.791 × 10^−18^	5.819 × 10^−18^	7.877 × 10^−18^

## Conclusion

In this paper, we provide four Windschitl type approximation formulas for the gamma function, and prove that those functions, involving the gamma function and Windschitl type functions, have good properties, including monotonicity and convexity. From these facts we obtain some new sharp Windschitl type bounds for the gamma and factorial functions. These sharp inequalities, together with numerical comparisons, illustrate that $W_{02} ( x ) $ defined by (1.9) is the best approximation formula among those mentioned in Sect. 5.

Moreover, we give a simple proof of Alzer’s inequalities (1.4), and improve and strengthen Lu et al.’s inequalities (1.6).

It is worth mentioning that our proofs of Theorems 1–5 are subtle and interesting, since the approximations deal with the gamma and hyperbolic sine functions, and it is difficult to establish their monotonicity and convexity by usual methods. Evidently, Lemmas 2 and 3 play important roles.
